# Sera proteomic features of active and recovered COVID-19 patients: potential diagnostic and prognostic biomarkers

**DOI:** 10.1038/s41392-021-00612-5

**Published:** 2021-06-03

**Authors:** Ling Leng, Mansheng Li, Wei Li, Danlei Mou, Guopeng Liu, Jie Ma, Shuyang Zhang, Hongjun Li, Ruiyuan Cao, Wu Zhong

**Affiliations:** 1 Stem cell and Regenerative Medicine Lab, Department of Medical Science Research Center, State Key Laboratory of Complex Severe and Rare Diseases, Translational Medicine Center, Peking Union Medical College Hospital, Peking Union Medical College and Chinese Academy of Medical Sciences, Beijing, 100730 China; 2 State Key Laboratory of Proteomics, Beijing Proteome Research Center, National Center for Protein Sciences (Beijing), Beijing Institute of Life Omics, Beijing, 102206 China; 3 National Engineering Research Center for the Emergency Drug, Beijing Institute of Pharmacology and Toxicology, Beijing, 100850 China; 4 Department of Infectious Diseases, Beijing YouAn Hospital, Capital Medical University, Beijing, 100069 China; 5 Department of Cardiovascular Surgery, Institute of Cardiac Surgery, PLA General Hospital, Beijing, 100853 China; 6 Department of Cardiology, Peking Union Medical College Hospital, Peking Union Medical College and Chinese Academy of Medical Sciences, Beijing, 100730 China; 7 Department of Radiology, Beijing YouAn Hospital, Capital Medical University, Beijing, 100069 China

**Keywords:** Systems biology, Predictive markers, Prognostic markers, Infectious diseases

**Dear Editor**,

The early diagnosis and prognosis of COVID-19 remain major challenges. At present, there are few studies focusing on the changes of sera proteome between active and recovered COVID-19 patients, and almost no biomarkers in sera are used to predict the function disorder of the injured tissues and organs in COVID-19 patients. In this study, we applied sera proteomics in the active and recovered COVID-19 patients, as well as healthy volunteers, combining with pathological staining of the patients’ tissues to identify the potential prognosis biomarkers that can reflect specific organ damage.

We collected sera samples from 32 patients with COVID-19, including 15 patients with both active and recovered samples, and 19 healthy donors (HDs) (Supplementary Table S1), and we used the data-independent acquisition (DIA) method to analyze these samples (Fig. 1a). After performing quality control analyses, 426, 455 and 460 proteins were identified in the sera from HDs, COVID-19 patients, and recovered COVID-19 patients, respectively (Fig. 1b, Supplementary Fig. S1, and Supplementary Table S2). There were 93 proteins differentially expressed (Supplementary Fig. S2a and Table S3) in the COVID-19 patients compared with the HDs. The proteins upregulated in COVID-19 patients were found to be involved in the inflammatory response, vesicle transport and immune response (Supplementary Fig. S2b and Fig. S3a–c). By contrast, the proteins downregulated in COVID-19 patients were found to be involved in the lipid-related processes, wound healing and coagulation (Supplementary Fig. S2b and Fig. S3d–f).

**Fig. 1:**
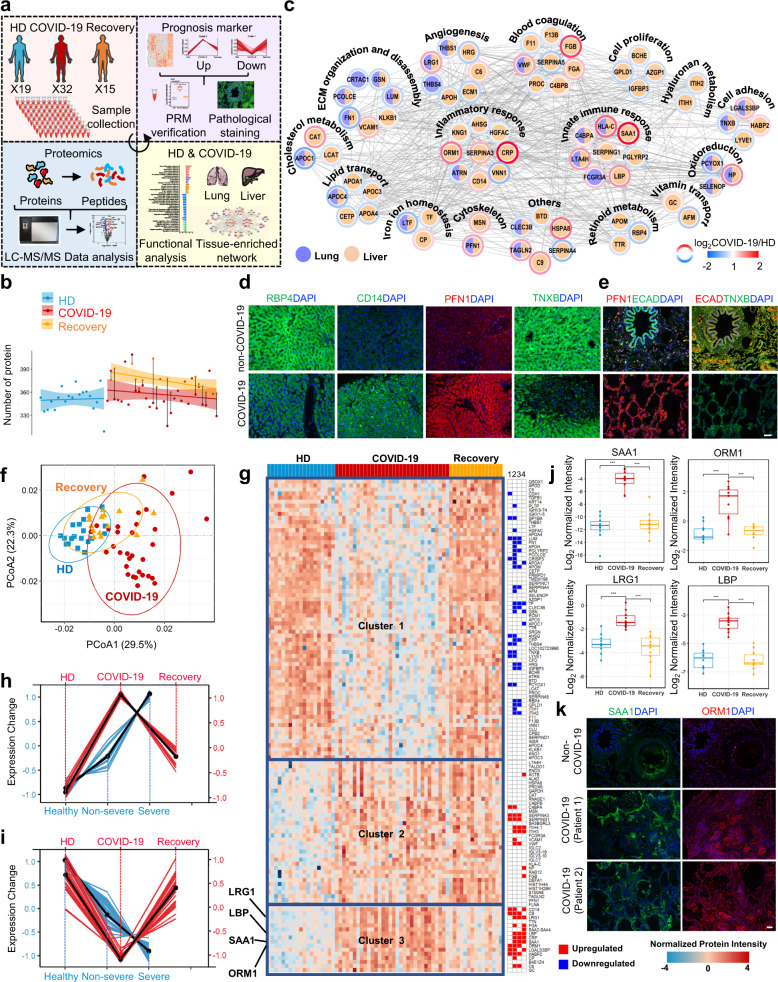
Functional characterization of diagnosis and prognosis biomarkers in sera of COVID-19 patients. **a** Schematic of the proteomics analysis used to evaluate sera from COVID-19 and recovered patients, and healthy donors (HDs). **b** The dashed curves fitted by linear regression show the distribution of protein identifications in the HD (blue, *n* = 19), COVID-19 patient (red, *n* = 32) and recovered COVID-19 patient (orange, *n* = 15) samples. The paired samples taken from COVID-19 patients during and after infection are annotated by straight black lines. The shading underneath the curves fit with lasso denotes the 95% confidence intervals. **c** Protein interaction network showing functional characterization of the differentially expressed sera proteins enriched in the liver and lung. Immunofluorescence analyses of RBP4, CD14, and PFN1 in liver tissue (**d**), and PFN1, ECAD, and TNXB in lung tissue (**e**) from COVID-19 patients and HDs (scale bars: 50 μm). **f** PCoA of quantitative proteome profiles of COVID-19 and recovered patients, and healthy individuals. **g** Heatmap showing the differentially expressed proteins in sera from COVID-19 and recovered patients, and HDs. Columns on the right represent the reported results (1, 2, 3 represent the differentially expressed proteins of Severe *vs*. Healthy, Non-severe *vs* Healthy, and Severe *vs*. Non-severe in Shen et al.’s work^3^; and 4 represents the differentially expressed proteins reported by Messner et al.^5^). Proteins (red lines) in Cluster 3 (**h**) and Cluster 1 (**i**) were compared with the reported data^3^ of patients with non-severe and severe COVID-19 and healthy individuals (blue lies). **j** Changes in the expression levels of selected differentially expressed proteins among HDs, active and recovered COVID-19 patients using a parallel reaction monitoring (PRM) strategy. Asterisks indicate statistical significance determined based on the Benjamini-Hochberg (BH)-adjusted *p* value from Limma’s pairwise comparison (*, <0.05; **, <0.01; ***, <0.001). **k** Immunofluorescence analyses of SAA1 and ORM1 in the lung tissue from patients with COVID-19 and HDs (scale bars: 50 μm)

We found that the differentially expressed proteins were enriched in various tissues, especially in liver and lung (Supplementary Fig. S4a, b), which are the target organs of SARS-CoV-2 infection.^1^ Then, we conducted the protein-protein interaction network of these lung- and liver-enriched differentially expressed proteins. Among the proteins enriched in liver, several proteins were involved in liver related functions, including the cell proliferation, retinoid, lipid and hyaluronan metabolism, vitamin transport, etc (Fig. 1c). These proteins included CD14 and RBP4. CD14, which is expressed in liver Kupffer cells, can be induced under lipopolysaccharide stimulation and is upregulated in inflammatory liver diseases. RBP4 is uniquely synthesized by hepatocytes, and its disorder is associated with several diseases, including retinal dystrophy, iris coloboma, comedogenic acne syndrome, and microphthalmia.^2^ The patients with COVID-19 had increased CD14 expression and decreased RBP4 expression (Fig. 1d), suggesting that these patients may have increased risk of inflammatory liver diseases and eye dysfunction.

Further, immunofluorescence staining of the lungs (Fig. 1e and Supplementary Fig. S4c) and livers (Fig. 1d and Supplementary Fig. S4c) showed decreased expression of tenascin XB, an essential regulator of collagen deposition by dermal fibroblasts, in patients with COVID-19, indicating an increased risk of lung/liver fibrosis in COVID-19 patients. Profilin-1 (PFN1), an actin-regulatory protein important in viral transcription activation and airway hyperresponsiveness, had been previously reported to be downregulated in non-severe COVID-19 patients compared to patients without COVID-19.^3^ By contrast, we found PFN1 to be upregulated not only in sera but also in tissues of the COVID-19 patients (Fig. 1d, e and Supplementary Fig. S4c), indicating the reliability of the biomarkers identified.

The principal coordinate analysis (PCoA) was performed, and the results indicated that the protein expression pattern in the recovered patients was closer to that in the HDs than that in the COVID-19 patients (Fig. 1f). This finding indicated that the sera molecular profile of recovered patients has returned to close to that of HDs. Then, unsupervised hierarchical clustering analysis (HCA) was performed, which resulted in the classification of the differentially expressed proteins among the three groups into three clusters (Fig. 1g and Supplementary Fig. S5). Cluster 1, consisting of the proteins that were downregulated in COVID-19 patients compared with HDs and recovered COVID-19 patients. These proteins could be used as prognosis biomarkers for monitoring the recovery process. Cluster 2, representing the upregulated proteins in the COVID-19 group that were not restored to the HDs levels in the recovered group, including antibodies, which were likely produced in response to SARS-CoV-2 infection. Among these antibodies, IGLC2, IGLC7, IGLV3-10, and IGLV3-19 had been previously reported to be upregulated in the B-cells of COVID-19 patients during COVID-19 pneumonia, and IGLC7 had been selected as a marker to distinguish severe and non-severe cases of COVID-19.^4^ Cluster 3, consisting of proteins upregulated in the COVID-19 patient compared to the HDs and recovered COVID-19 patient groups. As the expression of these proteins was upregulated after SARS-CoV-2 infection, while returned to HDs levels after recovery, they might be potential prognosis biomarkers.

We combined our dataset (proteins in Cluster 1 and 3 in Fig. 1g) with the published dataset,^3,5^ and we used HCA to identify trends in protein expression that might suggest COVID-19 severity (non-severe to severe COVID-19). The HCA results revealed two groups of proteins; the first group contained 23 sera proteins whose expression increased from HDs, patients with non-severe COVID-19, to patients with severe COVID-19 (Supplementary Fig. S6). Among them, 11 proteins were also included in Cluster 3 from our results (Fig. 1h). The second group contained 34 proteins whose expression decreased from HDs, patients with non-severe COVID-19 to patients with severe COVID-19 (Supplementary Fig. S6). Among them, 30 proteins were also included in Cluster 1 of our results (Fig. 1i). Next, we verified several of these potential biomarkers using a parallel reaction monitoring (PRM) strategy (Supplementary Table S4). The expression levels of the 10 proteins in the COVID-19 group were restored to the corresponding levels in HDs after disease recovery (Fig. 1j and Supplementary Fig. S7a), which further highlighted their values as prognostic biomarkers. Besides, the expression of three of these markers (SAA1, ORM1, and FGA) was confirmed in the lung (Fig. 1k) and/or liver (Supplementary Fig. S7b) tissues of COVID-19 patients using in situ histoimmunofluorescence. Among them, SAA1 and FGA were upregulated in the portal vein of liver, and CYP3A4 (a drug metabolizing enzyme, which is specifically expressed in central vein of liver) was low expressed in central vein of liver. Overall, these results suggested that the 41 proteins identified in these analyses might be prognostic biomarkers to monitor disease progression and can serve as quantifiable parameters to help evaluate therapeutic efficacy. In order to study whether the differentially expressed proteins in Cluster 1, 2 and 3 can indicate the disease caused by SARS-CoV-2 infection, different organ related diseases were analyzed (Supplementary Fig. S8). For example, proteins related to thrombosis (ORM1, CD14, FGA, etc.) in COVID-19 patients were upregulated, and central nervous system diseases (CETP, F13B, TF, etc.) were downregulated compared to those in recovered patients, indicating that the risk of these lung- and central nervous system-related diseases should be noted by health care providers during the treatment of COVID-19 patients.

In conclusion, this study has revealed a series of prognostic and diagnostic sera biomarkers of COVID-19, many of which are expressed in liver and lung tissues, and provided additional clues for the link between COVID-19 and multi-tissue/organ damages. More importantly, these biomarkers could provide some directions for rational evaluation of therapeutic efficacy and discovery of potential therapeutic drug targets of COVID-19.

## Supplementary information

Supplementary Materials

Supplementary Table S1

Supplementary Table S2

Supplementary Table S3

Supplementary Table S4

Supplementary Table S5

## Data Availability

All proteomics raw data have been deposited to the ProteomeXchange Consortium via the iProX partner repository with the dataset identifier PXD021954.
