# Supplementary material for: Sera proteomic features of active and recovered COVID-19 patients: potential diagnostic and prognostic biomarkers
Source: Signal Transduct Target Ther. 2021 Jun 3;6:216. doi: 10.1038/s41392-021-00612-5 (PMC8173321; doi:10.1038/s41392-021-00612-5)
Supplement: Supplementary file 1 — Supplementary Materials [file 41392_2021_612_MOESM1_ESM.pdf]

Supplementary Materials for

Sera proteomic features of active and recovered COVID-19 patients: potential diagnostic and prognostic biomarkers

Ling Leng†, Mansheng Li†, Wei Li†, Danlei Mou†, Guopeng Liu†, Jie Ma, Shuyang Zhang, Hongjun Li*, Ruiyuan Cao*, and Wu Zhong*

† These authors contributed equally.

* Correspondence authors: Hongjun Li (lihongjun00113@126.com), Ruiyuan Cao (21cc@163.com), or Wu Zhong (zhongwu@bmi.ac.cn).

This PDF file includes:

Supplementary Materials and Methods

Supplementary Figures S1 to S8

Legends for Supplementary Table S1 to S5

Other Supplementary Materials for this manuscript include the following:

Supplementary Table S1 to S5

Supplementary Materials and Methods

Human participants

Sera samples were obtained from 32 clinically confirmed COVID-19 patients diagnosed with COVID-19 according to the Chinese Government Diagnosis and Treatment Guideline (trial 5th version) (NHCPRC, 2020) at YouAn Hospital, Beijing, China (Supplementary Table S1) from January 31, 2020 to February 19, 2020, Laboratory confirmation of SARS-CoV-2 was performed at YouAn Hospital and the Academy of Military Medical Sciences, Beijing, China. To that end, throat swab specimens that had been obtained from all patients at admission were maintained in viral transport tube (KANGJIAN Medical Apparatus Co., Ltd, Jiangsu Province, China). RNA was assayed by using Real-Time Fluorescent RT-PCR kit for quantification of SARS-CoV-2 (Shenzhen BGI-GBI Biotech Co., Ltd, Shenzhen) according to the instruction, which was used for absolute quantification of ORF1ab gene. Additionally, all patients were evaluated by chest radiography and chest computed tomography, and histological staining of lung blocks was performed. We collected samples from patients who were classified with typical fever or respiratory tract symptoms with pneumonia based on the abovementioned guidelines (Supplementary Table S1). In addition, sera samples from 19 healthy individuals without COVID-19 were obtained from the Chinese PLA General Hospital from 2020.02.01 to 2020.02.15. Lung and liver tissues blocks with COVID-19 or without COVID-19 were obtained from YouAn Hospital. For the control blocks (without COVID-19), the healthy tissues (lungs and livers) were confirmed with no pathological features such as fibrosis and tumors. This study was approved by the Beijing YouAn Hospital Ethics Committee (NO. [2020]013), and written informed consent was obtained from the patients.

Immunofluorescence staining

Lung and liver tissue samples from patients with COVID-19 and HDs were washed twice with cold 1× PBS and fixed in a 10% neutral buffered formalin solution for 48 h at 4°C. After rinsing with cold 1× PBS, the samples were embedded in paraffin following standard protocols and sectioned at a thickness of 4 μm using a microtome for deparaffinization and rehydration. The sections were treated with 0.25% Triton X-100 for 20 min, blocked in 10% goat sera for 1 h at 25°C and incubated with primary antibodies overnight at 4°C. After incubation for 1 h at 25°C with secondary antibodies and counterstaining with DAPI, the sections were sealed with Fluoro-Gel for photography. Microscopy images were acquired at 20×/40× magnification and analysed by InForm (version 2.2). All equipments, reagents and supplies are available in Supplementary Table S5.

Protein extraction and tryptic digestion

Sera samples were inactivated at 95°C for 10 min. 50 μg of protein was lysed in a urea (UA) solution (8 M urea, 100 mM Tris-HCl, pH 8.0) with protease inhibitor. Then, 10 mM DTT was added to the samples and incubated for 4 h at 37°C. After centrifugation at 14,000g for 15 min at 25°C, 50 mM iodoacetamide (IAA) was added to the samples, which were incubated in the dark for 30 min at 25°C. Next, 25 mM NH_4HCO_3 was added to the samples, which were centrifuged for 10 min, which was repeated 4 times. Final digestion was performed at 37°C overnight by incubation of the samples with trypsin (1:50 enzyme:substrate). After centrifugation at 14,000 ×g for 30 min, the supernatants containing peptide mixtures were transferred to clean tubes for LC-MS/MS.

Mass spectrometry

The peptide mixtures were analysed using an Orbitrap Q-Exactive HF mass

spectrometer equipped with an Easy-nLC nanoflow liquid chromatography system. After drying, the peptides were resuspended in 0.1% formic acid and loaded onto a reverse chromatography column (150-μm inner diameter, XBridge peptide BEH C18, 1.9 μm; Waters Corp.). For the proteome profiling samples, peptides were separated on an analytical column over a 90-min gradient (buffer A: 0.1% formic acid and 99.9% H_2O; buffer B: 0.1% formic acid and 99.9% acetonitrile (ACN) at a constant flow rate of 0.5 μL/min (0-63 min, 6 to 22% buffer B; 63-77 min, 22% to 50% buffer B; 77-81 min, 50% to 90% buffer B; 81-90 min, 90% buffer B). A full mass spectrometry survey scan was carried out at a resolution of 60,000, the scan ranged from 300 to 1,500 m/z, the AGC target was 4e5, and the maximum ion injection time (max IT) was 50 ms. For the MS2 scan, the resolution was 15,000, charge state screening was enabled (precursor ions containing a charge of +2 to +7 were retained), and the isolation window was 1.6 m/z.

For data-dependent acquisition (DDA) MS runs, one full MS spectrum from 300 to 1500 m/z and 20 subsequent MS/MS scans were continuously acquired. The resolution for MS was set to 60,000, and that for MS/MS was set to 15,000. For high-energy collision dissociation (HCD), the isolation window was set to 1.6 m/z, and a normalized collision energy of 30% was applied. Dynamic exclusion for 20 s after the second fragmentation event was applied. We selected 25 proteins from Cluster 1 and Cluster 3 in Fig. 1f for parallel reaction monitoring (PRM) verification based on the results of the DIA experiment. The peptide mixtures were analysed using an Orbitrap Q-Exactive HF mass spectrometer equipped with an Easy-nLC nanoflow liquid chromatography system. For the proteome profiling samples, the peptides were separated on an analytical column over a 90-min gradient (buffer A: 0.1% formic acid and 99.9% H_2O; buffer B: 0.1% formic acid, 80% ACN and 19.9% H_2O) at a constant

flow rate of 0.6 μL/min (0-70 min, 7 to 30% buffer B; 70-85 min, 30% to 95% buffer B; 85-90 min, 95% buffer B). For a full mass spectrometry survey scan, the resolution was 60,000, the scan ranged from 300 to 1,500 m/z, the AGC target was 3e6, and the max IT was 80 ms. For the MS2 scan, the resolution was 15,000, charge state screening was enabled (to retain precursor ions containing a charge of +2 to +7), and the isolation window was 1.6 m/z.

Proteomic MS/MS data processing

The DDA data were processed with Proteome Discoverer (version 2.1, Thermo Fisher Scientific) and used to search against the UniProt human database (downloaded on 2019-7-31, containing 73,940 proteins). The parameters used for database searches were as follows: precursor and fragment mass tolerances of 10 ppm and 0.02 Da, respectively; trypsin as the digestion enzyme; a maximum number of missed cleavage sites of 2; oxidation (M) and acetylation (protein N-terminus) set as dynamic modifications; and the carbamidomethylation of cysteine set as a fixed modification. The identified proteins were filtered at both the PSM and protein levels by a 1% false discovery rate (FDR), which was determined by a target-decoy search strategy. All DDA results were loaded into Spectronaut (version 13.8.190930.43655, Biognosys, Switzerland) to generate the sample-specific spectral library. Then, the raw DIA data were processed on Spectronaut using the default settings. In brief, the retention time prediction type was set to dynamic iRT and correction factor for window. Mass calibration was set to local mass calibration. Decoy generation was set to scrambled (no decoy limit). Interference correction on the MS2 level was enabled, removing fragments for quantification based on the presence of interfering signals but maintaining at least three fragments for quantification. The FDR was estimated with the mProphet approach and set to 1% at the peptide level. Protein inference was

performed on the principle of parsimony using the ID Picker algorithm implemented in Spectronaut. To analyse the DIA runs with the spectral library, the RAW files were converted into the Spectronaut file format, after which the files were calibrated in the retention time dimension using the global spectral library. Subsequently, the recalibrated files were used for targeted data analysis with the spectral library without new recalibration of the retention time dimension.

A spectral library from PRM analysis was also constructed from the DDA data, and unique peptides of the target proteins were selected and exported to set the PRM method. The raw MS files from the PRM dataset were processed in Skyline (version 20.1.0.155). The top 5 product ions of target proteins in the library were used for comparison and quantification; the data were deemed reliable when the peak shape was intact and the retention time was within the set retention time range, and the undetected product ions were manually removed. Then, the peptide peak areas observed in samples from 15 COVID-19 patients and 15 HDs were exported into Excel for further analysis.

To identify useful prognostic biomarkers from the sera proteins of COVID-19 patients, we combined our dataset with the dataset published by Shen *et.al.*[1] The symptom classifications of the COVID-19 disease were according to the Chinese Government Diagnosis and Treatment Guideline (trial 5th version) (NHCPRC, 2020). The protein profiles of healthy, non-severe and severe samples generated in their research were downloaded ("Supplementary table mmc2.xlsx" in this published paper[1]). The proteins that overlapped with the differentially expressed proteins in HDs and COVID-19 patients in our study were kept for further statistics, heatmap visualization and hierarchical clustering analysis. The mean of protein intensities in each group samples were calculated and z-score normalized to represent the expression level of

the protein in the corresponding group. The analysis results are shown in Fig. 1g-i and Supplementary Fig. S6.

Statistical and Bioinformatics analysis

For proteomic analysis of the collected clinical samples, proteins simultaneously identified at least five times in the samples from healthy patients, patients with COVID-19 and recovered COVID-19 patients were maintained. The quantified values for expression of the remaining proteins were log2 transformed and z-score normalized. Then, the missing values for the remaining proteins were imputed with the minimum intensity of the protein identified in the same sample. To test for significant differences in the expression of proteins between HDs, patients with COVID-19 and recovered COVID-19 patients, multiple comparisons were performed with the R package Limma (version 3.38.3). Then, pairwise comparisons to determine the proteins whose expression was significantly different between pairs within the three experimental groups were performed using Limma as well. Differences for which Benjamini-Hochberg adjusted p-value < 0.01 were considered statistically significant.

The online tool DAVID (https://david.ncifcrf.gov/)[2] was used to annotate the proteins according to biological processes, cellular components, and molecular functions via Gene Ontology (GO)[3] analysis and assessed the enrichment of biological pathways in the differentially expressed proteins via Kyoto Encyclopedia of Genes and Genomes (KEGG)[4] pathway analysis. Tissue expression data for the proteins whose expression was significantly altered were retrieved from multiple repositories and integrated, including DAVID, Tissue 2.0,[5] HPA (www.proteinatlas.org) and UniProt (www.uniprot.org). A global protein interactome network for the proteins whose expression was differential between groups was built using Cytoscape (version

3.7.2),[6] and the protein-protein interactions were retrieved from the STRING database.[7] Principal coordinate analysis (PCoA) of the proteins whose values in each sample were valid was performed using the R package ape.[8] Volcano plots and heatmaps for the quantified values for proteins whose expression was significantly different between groups were produced using the R packages ggplot2 and ComplexHeatmap[9] (distance: Pearson, linkage: complete). We conducted all analyses and visualization using R statistical software version 3.3.2.

DATA AVAILABILITY

All proteomics raw data have been deposited to the ProteomeXchange Consortium via the iProX[10] partner repository with the dataset identifier PXD021954.

References

1. Shen, B. *et al.* Proteomic and Metabolomic Characterization of COVID-19 Patient Sera. *Cell* **182**, 59-72.e15 (2020).
2. Huang da, W., Sherman, B. T. & Lempicki, R. A. Bioinformatics enrichment tools: paths toward the comprehensive functional analysis of large gene lists. *Nucleic Acids Res.* **37**, 1-13 (2009).
3. Ashburner, M. *et al.* Gene ontology: tool for the unification of biology. The Gene Ontology Consortium. *Nat. Genet.* **25**, 25-29 (2000).
4. Ogata, H. *et al.* KEGG: Kyoto Encyclopedia of Genes and Genomes. *Nucleic Acids Res.* **27**, 29-34 (1999).
5. Palasca, O., Santos, A., Stolte, C., Gorodkin, J. & Jensen, L. J. TISSUES 2.0: an integrative web resource on mammalian tissue expression. *Database (Oxford)* **2018,** bay003 (2018).
6. Shannon, P. *et al.* Cytoscape: a software environment for integrated models of

biomolecular interaction networks. *Genome Res.* **13**, 2498-2504 (2003).

7. Szklarczyk, D. *et al.* The STRING database in 2017: quality-controlled protein-protein association networks, made broadly accessible. *Nucleic Acids Res.* **45**, D362-D368 (2017).

8. Paradis, E. & Schliep, K. ape 5.0: an environment for modern phylogenetics and evolutionary analyses in R. *Bioinformatics* **35**, 526-528 (2019).

9. Gu, Z., Eils, R. & Schlesner, M. Complex heatmaps reveal patterns and correlations in multidimensional genomic data. *Bioinformatics* **32**, 2847-2849 (2016).

10. Ma, J. *et al.* iProX: an integrated proteome resource. *Nucleic Acids Res.* **47**, D1211-D1217 (2019).

Supplementary Figures

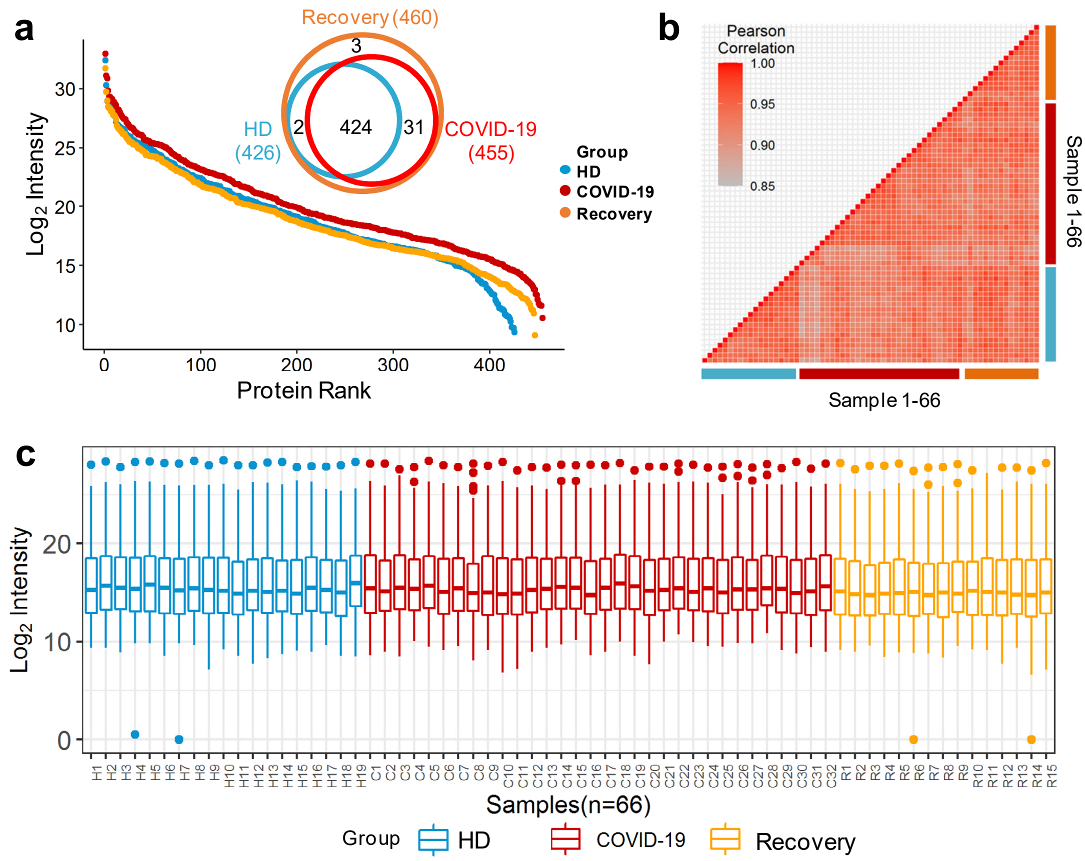

Fig. S1 Quality control analyses of proteomics datasets. **a** Dynamic ranges of the sera proteomes of the HD, COVID-19 patient, and recovered COVID-19 patient samples. Venn diagram showing the numbers of proteins in the HD, COVID-19, and recovered patient sera samples and the number of common proteins. **b** Heatmap showing the pairwise Pearson's correlation coefficient between the HD, COVID-19 patient and recovered COVID-19 patient samples (range: 0.85-1.00). **c** Distribution of the $\log_2$ intensities of the proteins identified from 66 proteome samples. Blue represents HD samples (*n*=19), red represents COVID-19 patient samples (*n*=32), and orange represents recovered COVID-19 patient samples (*n*=15). In the box plots, the middle bar represents the median, and the box represents the interquartile range; bars extend to 1.5× the interquartile range; and the dots represent the outlier values of protein expression.

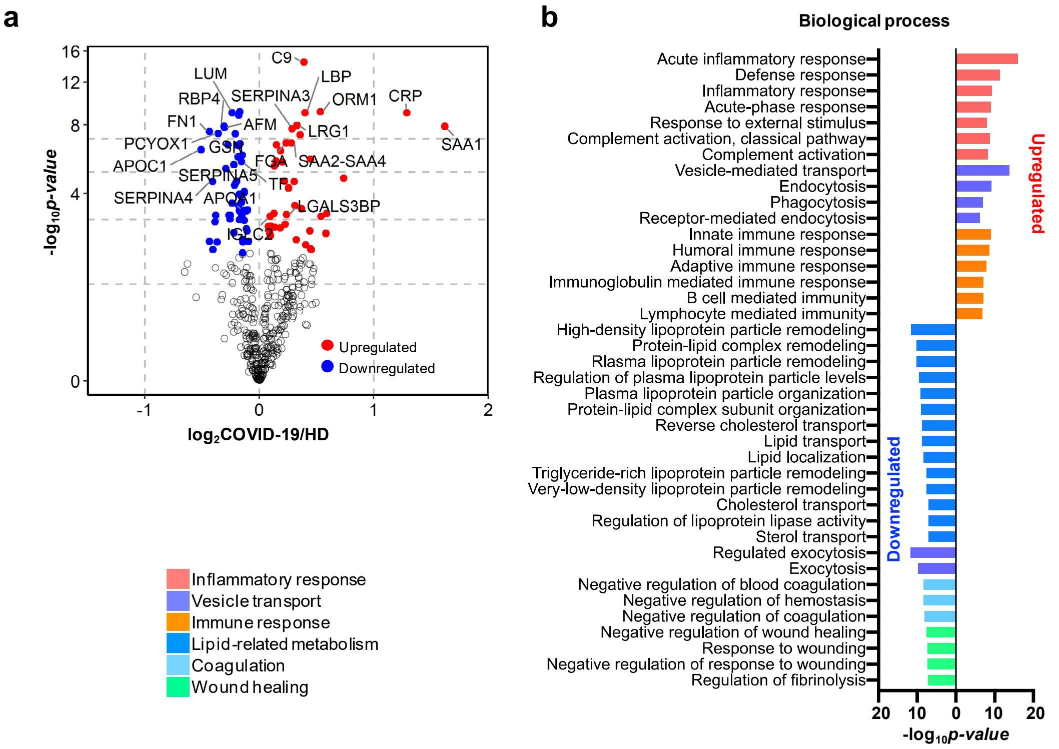

Fig. S2 Quantitative proteomic profiles and pathological features in the sera of patients with COVID-19 and healthy patients. **a** Volcano plot of the $-\log_{10}$ p-value *vs.* the $\log_2$ protein abundance in sera from healthy patients and from patients with COVID-19. Proteins outside the significance threshold lines ($-\log_{10} p\text{-}value > 2$) are shown in red (upregulated) or blue (downregulated). The p-values were calculated by the Benjamini-Hochberg (BH)-adjusted *p*-values from pairwise comparison of the proteins identified in sera from healthy patients *vs.* COVID-19 patients with Limma. **b** Biological process analysis of differentially expressed proteins in the sera of patients with COVID-19 *vs.* healthy patients ranked according to $\log_{10}$ *p-value*. Colours indicate the functional categories.

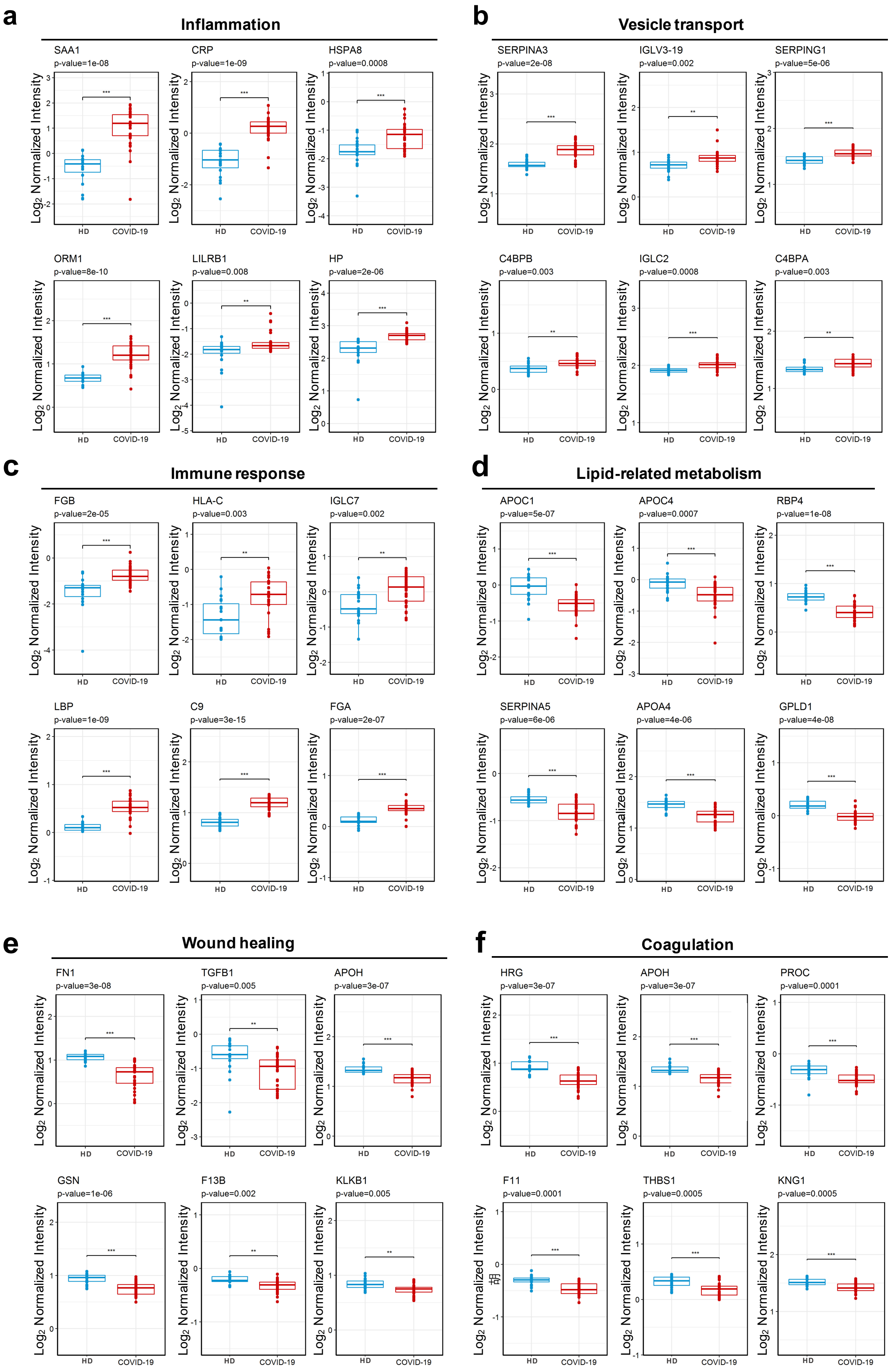

Fig. S3 Dysregulated sera proteins in COVID-19. Expression level changes (z-score-normalized log2-transformed Intensity) of selected functional proteins that

are significantly upregulated (**a-c**) and downregulated (**d-e**) between HDs and COVID-19 patients. The blue and red dots represent the sera samples of the HDs and COVID-19 patients, respectively. Asterisks indicate statistical significance determined based on the Benjamini-Hochberg (BH)-adjusted *p-value* from pairwise comparison with Limma. BH-adjusted *p-value*: *, < 0.05; **, < 0.01; ***, < 0.001.

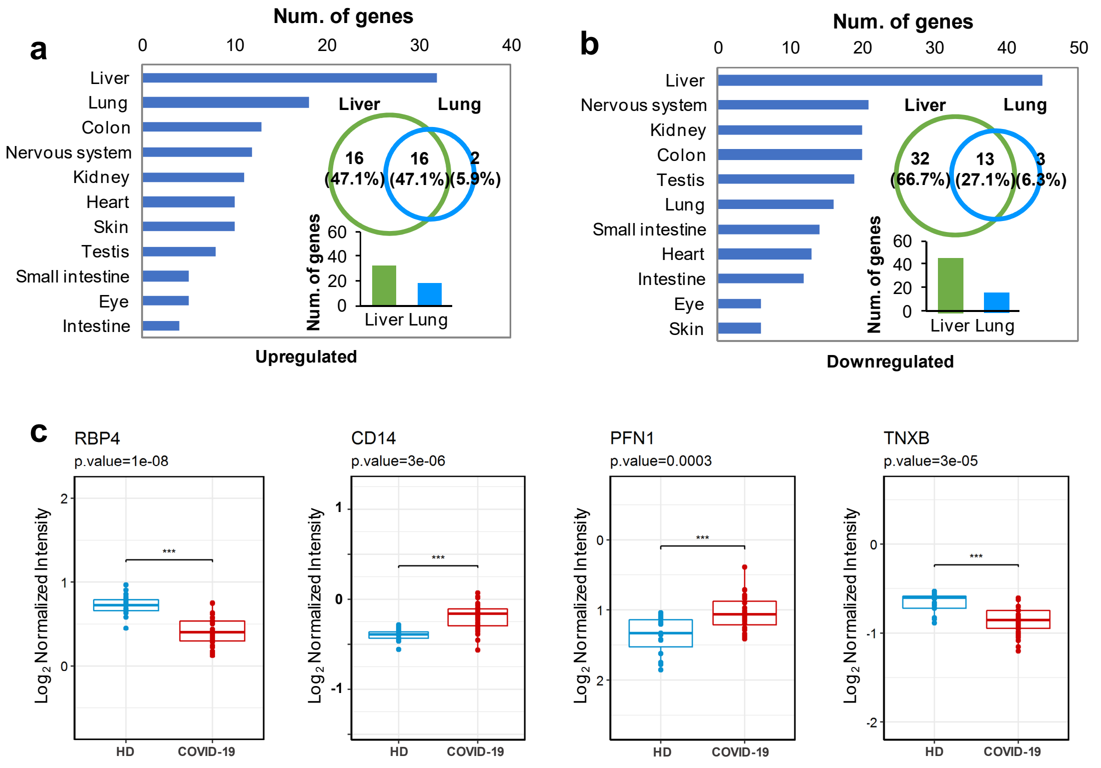

Fig. S4 Tissue expression analysis of dysregulated sera proteins in COVID-19. The upregulated proteins (**a**) and downregulated proteins (**b**) in COVID-19 patients compared to healthy patients enriched in 11 tissues/systems, including the liver, lung, colon, kidney, heart, skin, testis, small intestine, eye, intestine and nervous system. Venn diagrams showing the number and proportion of dysregulated sera proteins in the liver and lung. The differentially expressed proteins, including the upregulated and downregulated proteins, were filtered by the Benjamini-Hochberg adjusted *p*-value (< 0.01) of Limma's pairwise comparisons in the COVID-19 patients with the HDs. **c** Expression level changes (z-score-normalized log2-transformed Intensity) of RBP4, CD14, PFN1, and TNXB among HDs and active and recovered COVID-19 patients.

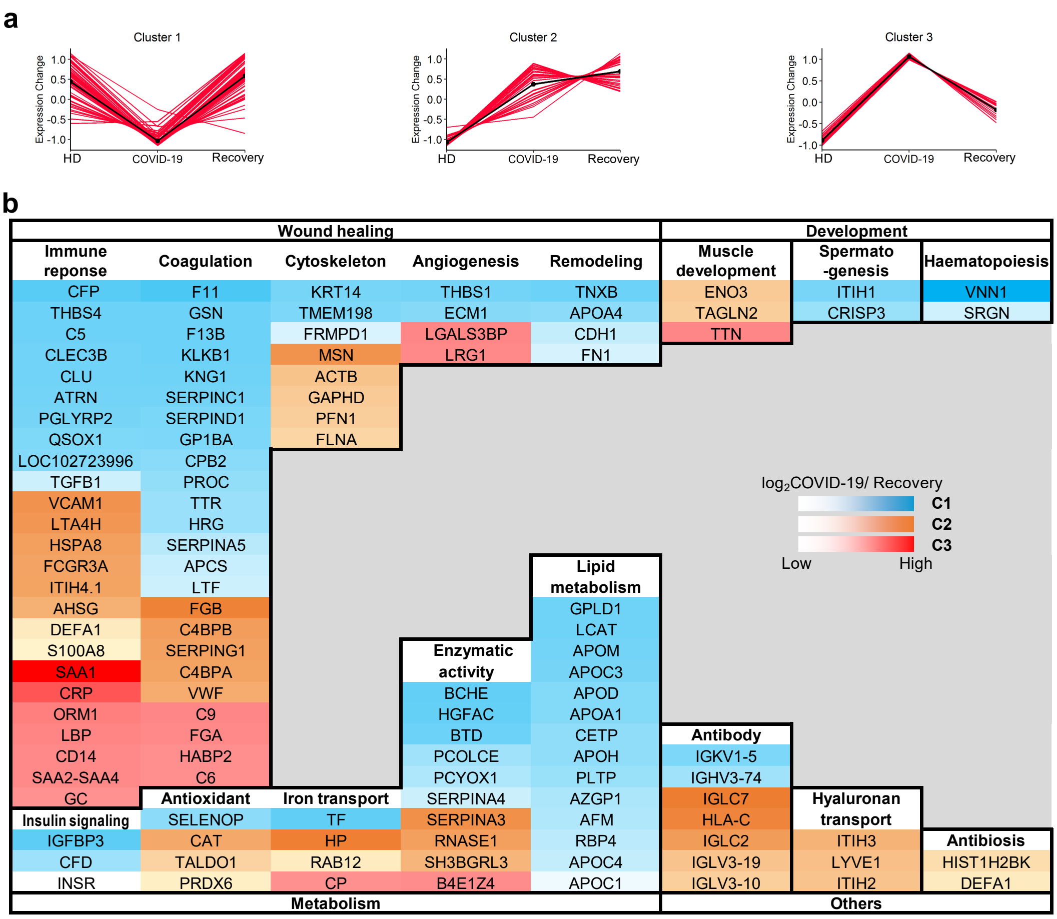

Fig. S5 Functional analysis of the differentially expressed sera proteins among HDs and active and recovered COVID-19 patients. **a** The co-expression patterns of the proteins in the three modules (cluster 1, cluster 2 and cluster 3) that presented in the Fig. 1f. **b** Heatmap analyisis of the expression patterns of the differentially expressed sera proteins among HDs and active and recovered COVID-19 patients. Blue (C1, Cluster 1, differentially expressed proteins that are downregulated in COVID-19 patients compared to HDs and recovered COVID-19 patients), orange (C2, Cluster 2, differentially expressed proteins that are upregulated in COVID-19 patients and not restored to baseline levels in recovered COVID-19 patients) and red (C3, Cluster 3, differentially expressed proteins that are upregulated in COVID-19 patients compared to HDs and recovered COVID-19 patients) boxes indicate the $\log_2$ fold changes of the proteins intensities among active and recovered COVID-19 patients.

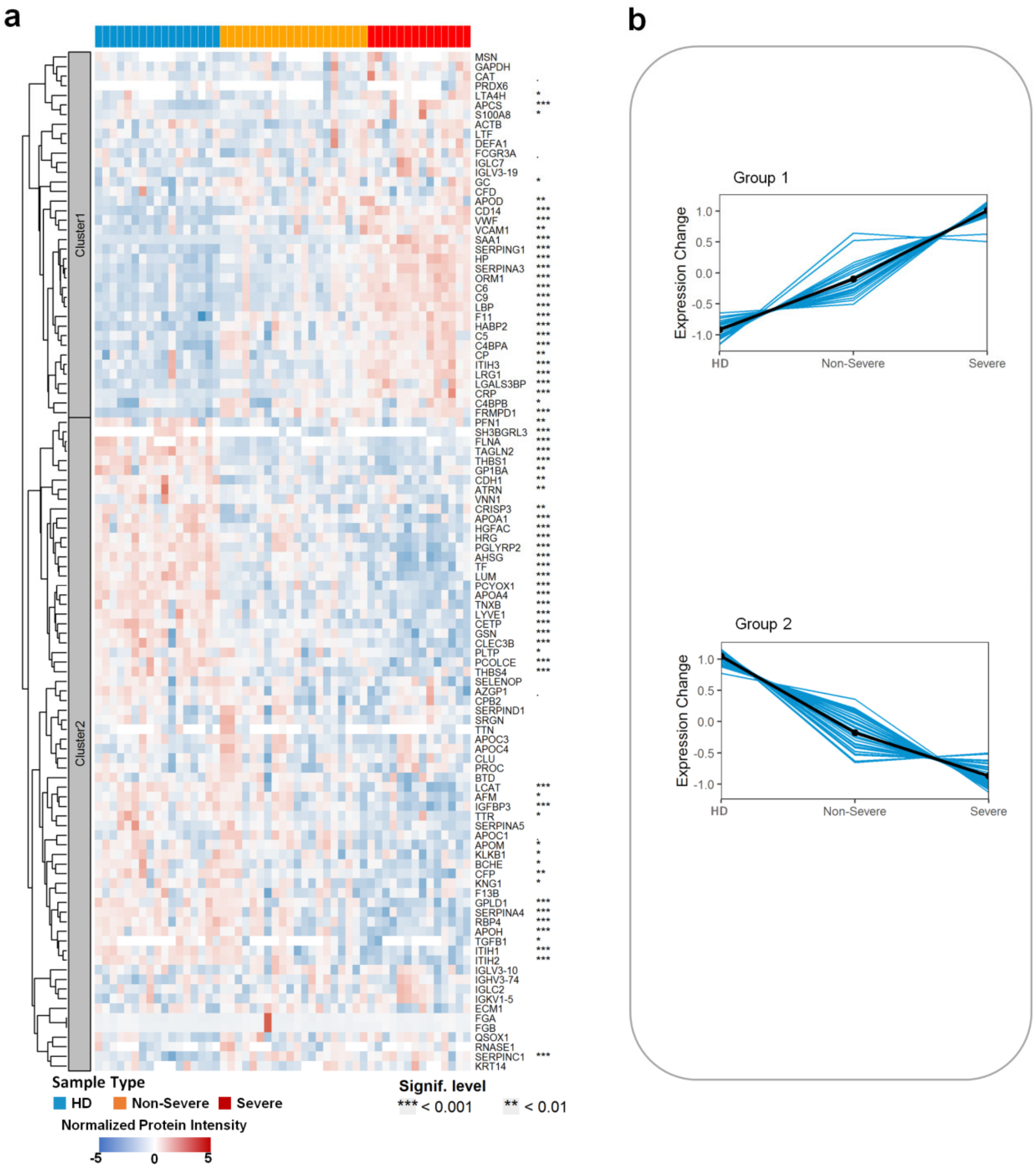

Fig. S6 Dysregulated proteins in the sera of non-severe and severe COVID-19 patients. **a** Heatmap showing proteins identified from the sera of healthy and patients with non-severe and severe COVID-19 (the raw data were published by Shen *et al.*[1]) and found to be differentially expressed among HDs and active and recovered COVID-19 patients in this study. Red and blue boxes indicate the row z-scored values of the intensities of upregulated and downregulated proteins, respectively. Asterisks indicate statistical significance determined based on the Benjamini-Hochberg (BH)-adjusted *p*-value from multiple comparison with Limma. BH-adjusted *p-value*:

*, < 0.05; **, < 0.01; ***, < 0.001. **b** The co-expression patterns of the proteins in the two modules (Cluster 1 and Cluster 2) are presented. Cluster 1 and Cluster 2 represent the enriched sera proteins that are gradually upregulated and downregulated, respectively, with the progression from non-severe to severe disease.

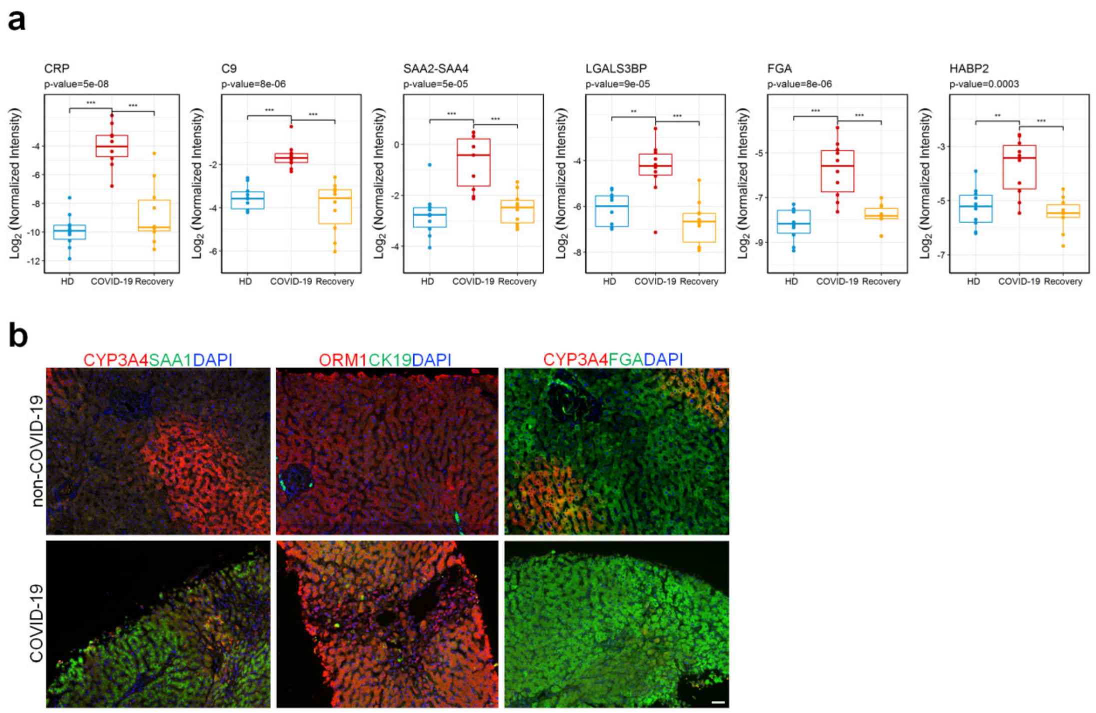

Fig. S7 Verification of dysregulated sera proteins in COVID-19. **a** Changes in the expression levels (normalized log2-transformed intensity) of selected proteins whose expression significant differed among samples from HDs and active and recovered COVID-19 patients using a parallel reaction monitoring (PRM) strategy. Asterisks indicate statistical significance determined based on the Benjamini-Hochberg (BH)-adjusted *p-value* from Limma's pairwise comparison. BH-adjusted *p-value*: *, < 0.05; **, < 0.01; ***, < 0.001. **b** Immunofluorescence analyses of CYP3A4, SAA1, ORM1, CK19, and FGA in liver tissue from patients with COVID-19 and healthy patients (scale bars: 50 μm).

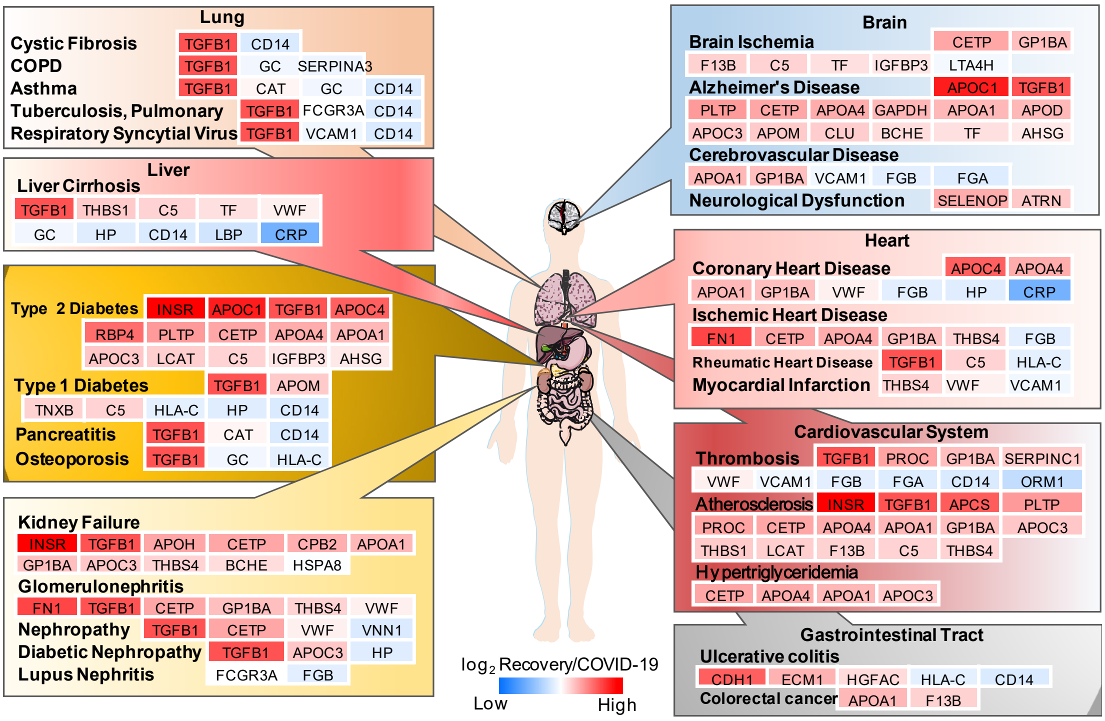

Fig. S8. Schematic of differentially expressed proteins in diseases related to different tissues and organs in recovered patients compared to those in patients with COVID-19. Red and blue boxes indicate the log2 protein abundance in sera from recovered patients and from patients with COVID-19.

Legends for Supplementary Table S1 to S5

Table S1-1. Overview of the characteristics of patients diagnosed with COVID-19 involved in the study.

Table S1-2. Overview of the characteristics of healthy donors involved in the study.

Table S2. All Proteins identified in sera samples of active and recovered COVID-19 patients and healthy donors (HDs).

Table S3-1. Differentially expressed proteins between HDs and COVID-19 patients sera samples.

Table S3-2. Differentially expressed proteins between COVID-19 and recovered COVID-19 patients sera samples.

Table S3-3. Differentially expressed proteins among HDs, COVID-19 and recovered COVID-19 patients sera samples.

Table S4. Detailed information about the biomarkers validated by PRM strategy.

Table S5. Companies providing equipment, reagents and/or supplies.
